# Delayed Presentation of Pityriasis Rubra Pilaris in a Patient on Treatment With Ponatinib

**DOI:** 10.7759/cureus.52155

**Published:** 2024-01-12

**Authors:** Marissa M Yaldo, Hailey Olds, Meredith Hengy, Meena Moossavi

**Affiliations:** 1 School of Medicine, Wayne State University, Detroit, USA; 2 Department of Dermatology, Wayne State University School of Medicine, Detroit, USA

**Keywords:** delayed presentation, adverse drug reactions, pityriasis rubra pilaris, ponatinib, chronic myeloid leukemia

## Abstract

Pityriasis rubra pilaris (PRP) is a rare dermatologic condition whose etiology is largely unknown. However, some medications, including ponatinib, have been implicated. Our case features an 80-year-old patient who developed PRP after two-and-a-half years of ponatinib use. We present this case due to the rare presentation of ponatinib-induced PRP as well as its significantly delayed presentation.

## Introduction

Pityriasis rubra pilaris (PRP) is a rare condition that causes inflammation, scaling, and thickening of the skin [1]. The etiology of PRP is largely unknown. Some cases are known to be familial, following mainly an autosomal dominant inheritance pattern with a gain of function mutation in the CARD14 gene. However, the majority of PRP cases are sporadic and have been associated with infections, UV exposure, trauma, and autoimmune conditions such as myositis and celiac disease. In rare cases, medications such as imatinib and ponatinib have been implicated [1]. There have been 12 reported cases of ponatinib-induced PRP to date, and here we present the thirteenth case with a significantly delayed presentation [2,3].

## Case presentation

**Figure 1:**
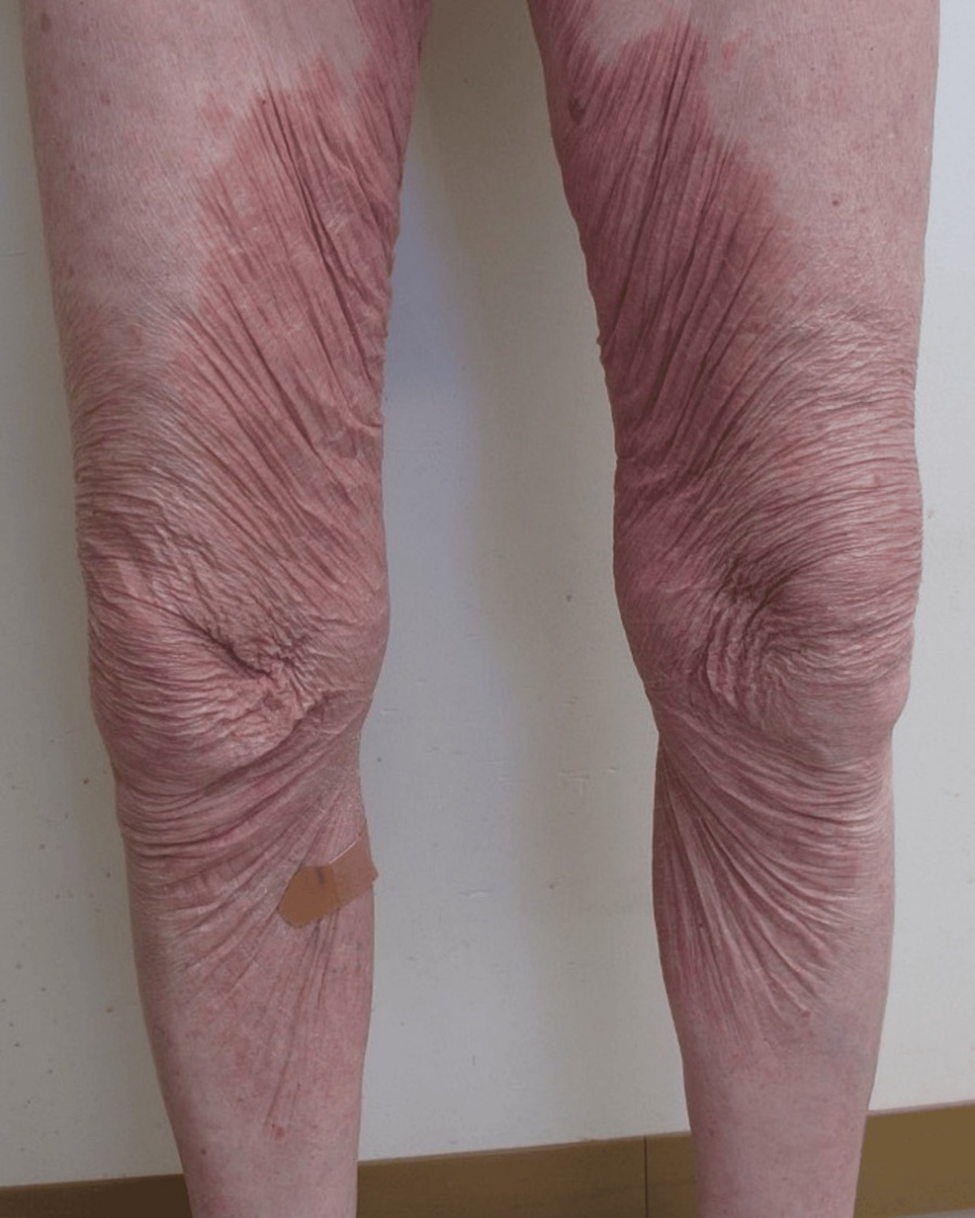
Appearance of red-orange, well-defined plaques of bilateral lower extremities as well as significant postinflammatory cutis laxa of the skin

**Figure 2:**
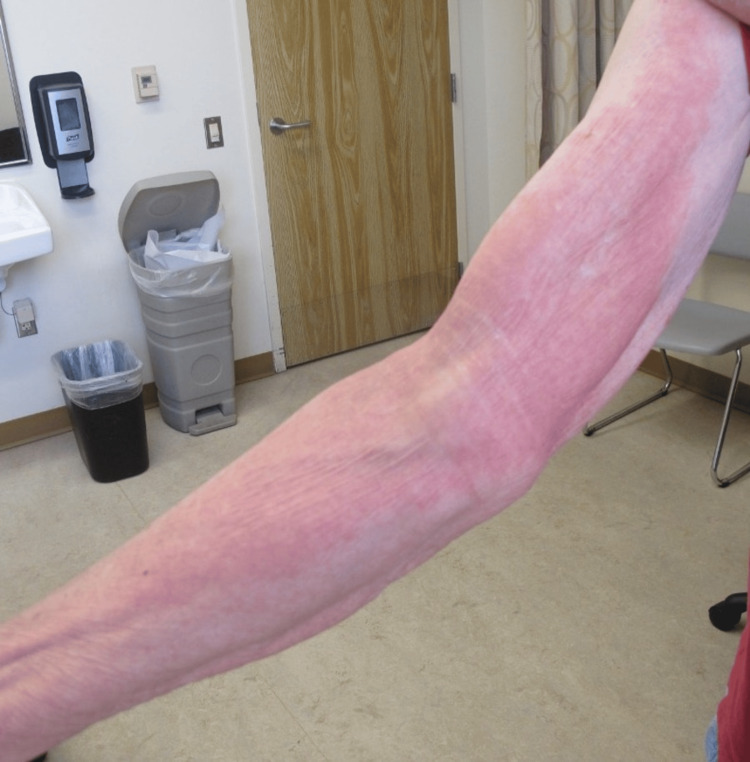
Appearance of well-defined erythematous coalescing plaques on the right upper extremity.

An 80-year-old patient presented with a one-month history of a rash on his bilateral upper and lower extremities. He denied pruritus, pain, and tenderness. He also denied any trigger and noted the rash was resolving at the time of his visit. The only attempted treatment was ammonium lactate cream. He denied any new personal care products or medications. Of note, the patient was actively being treated for chronic myeloid leukemia (CML) with ponatinib, which was started about two-and-a-half years prior. Prior to this, his CML was treated with dasatinib, bosutinib, and hydroxyurea. Physical exam revealed well-defined, red-orange plaques on the bilateral upper and lower extremities (Figure 1) with an overlying scale around the elbows (Figure 2) as well as post-inflammatory cutis laxa. There was also involvement of the back and abdomen. Islands of sparing were present. His palms, chest, scalp, and nails were largely uninvolved.

**Figure 3:**
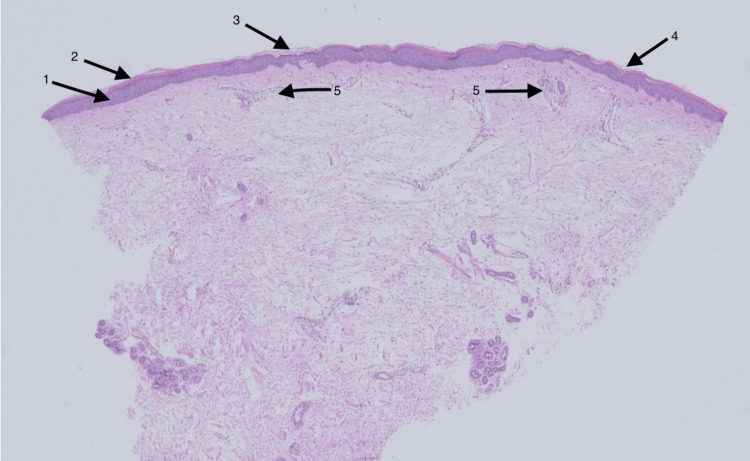
Punch biopsy stained with hematoxylin-eosin at 4x magnification, demonstrating alternating parakeratosis and orthokeratosis vertically and horizontally, acanthosis, follicular plugging, and scant perivascular lymphohistiocytic infiltrate Arrow 1: irregular acanthosis; Arrow 2: alternating parakeratosis; Arrow 3: orthokeratosis (arrow 3); Arrow 4: follicular plugging; Arrow 5: scant lymphocytic infiltrate

A punch biopsy was obtained from the forearm and lower leg. Histopathological findings revealed irregular acanthosis (arrow 1), alternating parakeratosis (arrow 2) with orthokeratosis (arrow 3) vertically and horizontally, follicular plugging (arrow 4), and scant lymphocytic infiltrate (arrow 5), as can be seen in Figure 3. With these findings, as well as a careful drug history, a diagnosis of PRP was made. Differential diagnoses included drug-induced erythema, morbilliform eruption, atopic dermatitis, and psoriasis. However, these were excluded given the histopathologic correlation and drug history. Though the rash was asymptomatic, the patient wanted to treat its appearance. Therefore, triamcinolone 0.1% ointment twice daily was initiated. Ponatinib was continued for his CML treatment. The patient's rash slowly improved after a few months of topical steroid therapy.

## Discussion

Ponatinib is an oral tyrosine kinase inhibitor that blocks BCR-ABL1 to treat refractory CML or Philadelphia chromosome-positive acute lymphoblastic leukemia (Ph(+) ALL) [4]. Ponatinib can also block the activity of other kinases such as fibroblast growth factor, platelet-derived growth factor, vascular endothelial growth factor, KIT, FMS like tyrosine kinase 3, and SRC families. Further, ponatinib’s diverse spectrum of targets may inadvertently involve the skin [5] and, accordingly, a common side effect of treatment is a rash. According to the phase 2 Ponatinib Ph(+) ALL and CML Evaluation (PACE) trial, 47% of CML patients treated with ponatinib developed a rash [4]. Rashes caused by tyrosine-kinase inhibitors have been described as maculopapular, lichenoid, psoriasiform, and dyschromic [6]. Rarely, a PRP-like eruption occurs, and underlying conditions, such as hepatitis C infection, type I diabetes, and malignancies, may put individuals at an increased risk for the development of PRP [7]. The mean onset time of the PRP-like reaction from starting ponatinib is five weeks, but here, we present a unique case of delayed presentation that began about two-and-a-half years after initiating ponatinib [6].

Several studies indicate a delay in the correct diagnosis of PRP, so it is highly important to understand its presentation in order to make a timely diagnosis and initiate proper treatment [8,9]. In adults, there are two main subtypes of PRP: type I, which is characterized by classic red-orange plaques with islands of sparing, perifollicular keratotic papules, and palmoplantar keratoderma, and type II, which is characterized by areas of eczematous dermatitis, scale on the legs, and keratoderma with a coarse scale [1]. Most cases of ponatinib-induced PRP are classified as type II, and, accordingly, our patient’s presentation is more consistent with type II [10]. In most cases of tyrosine kinase inhibitor-induced PRP, there is an absence of the classic palmoplantar keratoderma, which aligns with this patient’s presentation [6]. Of note, there are additional subtypes of PRP, including type III, type IV, and type V, which all primarily occur in children [11]. The latest addition to the categorization of PRP is type VI, which is related to human immunodeficiency virus infection and is associated with a poor prognosis [12].

Symptoms such as pruritus and pain as well as skin appearance can be treated with topical steroids or retinoids, and most patients are able to continue ponatinib therapy for the necessary treatment of their leukemia. Oral corticosteroids, retinoids, and biologics have also been successfully used for more generalized cases [1]. Interleukin-23, interleukin-17, and tumor necrosis factor inhibitors have demonstrated superior efficacy in rash clearance [8]. However, overall time to resolution is variable [1]. Traditionally, complete resolution may take anywhere from two to three years [13], but recent studies demonstrate persistence well beyond this timeframe [9].

## Conclusions

Due to the varied etiology of PRP, it is important to gather a detailed medical history when making the diagnosis. Pertinent medical history should include a history of trauma, recent or chronic infections, family history, and personal history of autoimmune conditions. A detailed medication reconciliation should also be performed, paying special attention to drugs such as imatinib and ponatinib. Clinicians should consider the possibility of PRP in patients who are taking ponatinib, even if it has been several months or years since initiating the drug. A biopsy may be useful to confirm the diagnosis. Finally, ponatinib and imatinib are usually essential components of leukemia treatment regimens and should not be discontinued, as most cases of PRP do not cause significant morbidity and mortality.
